# Return to work after hospitalization for sepsis: a nationwide, registry-based cohort study

**DOI:** 10.1186/s13054-023-04737-7

**Published:** 2023-11-15

**Authors:** Nina Vibeche Skei, Karoline Moe, Tom Ivar Lund Nilsen, Lene Aasdahl, Hallie C. Prescott, Jan Kristian Damås, Lise Tuset Gustad

**Affiliations:** 1 Department of Intensive Care and Anesthesia, Nord-Trondelag Hospital Trust, Levanger, Norway; 2 The Mid-Norway Centre for Sepsis Research, Institute of Circulation and Medical Imaging, Norwegian University of Science and Technology (NTNU), Trondheim, Norway; 3 Department of Public Health and Nursing, Norwegian University of Science and Technology (NTNU), Trondheim, Norway; 4 Unicare Helsefort Rehabilitation Centre, Rissa, Norway; 5 Department of Internal Medicine, University of Michigan, Ann Arbor, MI USA; 6 VA Center for Clinical Management Research, Ann Arbor, MI USA; 7 Centre of Molecular Inflammation Research, Institute for Clinical and Molecular Medicine, Norwegian University of Science and Technology (NTNU), Trondheim, Norway; 8 Department of Infectious Diseases, St. Olav’s University Hospital, Trondheim, Norway; 9 Faculty of Nursing and Health Sciences, Nord University, Levanger, Norway; 10 Department of Medicine and Rehabilitation, Levanger Hospital, Nord-Trøndelag Hospital Trust, Levanger, Norway

**Keywords:** Sepsis, RTW, ICU, COVID-19

## Abstract

**Background:**

Sepsis survivors commonly experience functional impairment, which may limit return to work. We investigated return to work (RTW) of patients hospitalized with sepsis and the associations with patient and clinical characteristics.

**Methods:**

Working-age patients (18–60 years) admitted to a Norwegian hospital with sepsis between 2010 and 2021 were identified using the Norwegian Patient Registry and linked to sick-leave data from the Norwegian National Social Security System Registry. The main outcome was proportion of RTW in patients hospitalized with sepsis at 6 months, 1 year, and 2 years after discharge. Secondary outcomes were time trends in age-standardized proportions of RTW and probability of sustainable RTW (31 days of consecutive work). The time trends were calculated for each admission year, reported as percentage change with 95% confidence interval (CI). Time-to-event analysis, including crude and adjusted hazard risk (HRs), was used to explore the association between sustainable RTW, characteristics and subgroups of sepsis patients (intensive care unit (ICU) vs. non-ICU and COVID-19 vs. non-COVID-19).

**Results:**

Among 35.839 hospitalizations for sepsis among patients aged 18–60 years, 12.260 (34.2%) were working prior to hospitalization and included in this study. The mean age was 43.7 years. At 6 months, 1 year, and 2 years post-discharge, overall estimates showed that 58.6%, 67.5%, and 63.4%, respectively, were working. The time trends in age-standardized RTW for ICU and non-ICU sepsis patients remained stable over the study period, except the 2-year age-standardized RTW for non-ICU patients that declined by 1.51% (95% CI − 2.22 to − 0.79) per year, from 70.01% (95% CI 67.21 to 74.80) in 2010 to 57.04% (95% CI 53.81–60.28) in 2019. Characteristics associated with sustainable RTW were younger age, fewer comorbidities, and fewer acute organ dysfunctions. The probability of sustainable RTW was lower in ICU patients compared to non-ICU patients (HR 0.56; 95% CI 0.52–0.61) and higher in patients with COVID-19-related sepsis than in sepsis patients (HR 1.31; 95% CI 1.15–1.49).

**Conclusion:**

Absence of improvement in RTW proportions over time and the low probability of sustainable RTW in sepsis patients need attention, and further research to enhance outcomes for sepsis patients is required.

**Supplementary Information:**

The online version contains supplementary material available at 10.1186/s13054-023-04737-7.

## Introduction

Sepsis is caused by a dysregulated host immune response to infection, resulting in acute organ dysfunction [1], and is a major cause of worldwide morbidity and mortality, with an estimated 50 million cases and 11 million sepsis-related deaths in 2017 [2]. Sepsis survivors often experience poor long-term outcomes with new or worsened cognitive and functional impairments [3, 4], making normal activities hard to resume. However, the impact of these problems on sepsis survivors’ ability to return to work (RTW) is less clear.

RTW is a recommended measure of the long-term functional level after disease, including trauma [5], acute respiratory distress syndrome (ARDS), and conditions requiring intensive care and organ support [6–10]. A previous Danish study (2018) investigating a general intensive care unit (ICU) cohort found that among survivors receiving organ support therapy 60% had returned to work at 1 year and 68% at 2 years after discharge [9]. Prior studies suggest that sepsis survivors have worse overall functional outcomes than other intensive care survivors [11, 12]. This is supported by a recent administrative-based German study (2023) investigating RTW, which found that only 55% and 65% of ICU-treated sepsis patients returned to work 6 months and 1 year after discharge [13]. However, negative health impact can persist over years [10] and warrant RTW estimates beyond 1 year. Previous estimates were based on the Sepsis-1 and Sepsis-2 definition [13]. Thus, there is a need for updated estimates based on the latest Sepsis-3 definition to monitor prognosis and trends over time to plan appropriate interventions for sepsis survivors working prior to admission.

Considering the recent pandemic, research on reduced functioning after infection with COVID-19 is evolving, suggesting that 30% of survivors are affected [14–17]. RTW estimates at 6 months after a COVID-19 admission vary between 57% and 89% [18–21]. Currently, limited research is available on RTW for patients with COVID-19-related sepsis, but one study of 120 COVID-19 patients found no differences in self-reported RTW after 110 days between non-ICU and ICU patients [22].

In sum, knowledge about long-term outcomes is warranted to understand and facilitate the RTW process for sepsis patients [23]. The main aim of this nationwide registry study was therefore to investigate the RTW proportion in patients admitted with sepsis, including ICU, non-ICU and COVID-19 sepsis, at 6 months, 1 year, and 2 years after discharge in the period from 2010 through 2021. The secondary aims were to examine temporal trends in RTW and investigate characteristics associated with sustainable RTW, defined as working at least 31 consecutive days after a hospital discharge with an index sepsis episode.

## Material and method

### Design and setting

In this Norwegian nationwide registry-based study, we identified hospitalizations for sepsis using ICD-10 codes in the Norwegian Patient Registry (NPR) [24]. To identify ICU sepsis patients, we linked NPR individual-level data with the Norwegian Intensive Registry (NIR) [25]. Reporting to NPR and NIR is mandatory, and the linkage was complete. For more information about the registries used in this study, see Additional file 1: File S1.  

The Norwegian adult population in working age (18–60 years) was 2.735.188 in 2010, which increased to 3.004.285 in 2021 [26]. The population have access to public healthcare that covers all emergency incidents and is free of charge at the point of delivery. Private hospitals are for some outpatients and provide non-emergency treatment, and not used for severely ill patients like those needing acute hospitalization due to sepsis.

#### The Norwegian National Insurance Scheme

The employment rate in Norway in the last quarter of 2022 was 80.2%, while 2.4% was unemployed and 17.8% were defined as outside the workforce. 77.7% of all women in are employed, and of those 72.1% works fulltime. Self-employed workers make up 4.5% of the total employment. 9.5% of the workforce receives fully or partly disability pension, where 63.0% of these are under 60 years of age, and the majority are women (58%) [26].

All Norwegian workers have a compulsory membership in The Norwegian National Insurance Scheme [27]. Individuals who have been working for at least four weeks before illness, with an income higher than ½ of the ‘basic amount’ (NOK 118.620, or USD 11.798 in 2023), and who have lost work income because of a medically certified illness are entitled to sickness benefits of up to 52 weeks. Sickness benefits begin on the day the employer is notified of the illness. Self-employed individuals and freelance workers are also entitled to benefits, but must cover the first 16 days of absence themselves. After 52 weeks, it is possible to apply for more long-term medical benefits, work assessment allowance and permanent disability pension. To qualify for a disability pension, individuals must have at least a 50% reduction in workability documented by a doctor's certificate. A membership of The Norwegian National Insurance Scheme qualifies for a medical benefit application, even though the patient is without sickness benefits rights. All individuals with benefits in Norway are registered by their social security number in the Norwegian National Social Security System Registry, run by The Norwegian Labour and Welfare Administration [28].

### Study population

We included patients from all Norwegian public hospitals in the period from 2010 through 2021 with an index admission for sepsis, defined by an ICD-10 code for infection in combination with an ICD-10 code for acute organ dysfunction (implicit) and/or an ICD-10 code for specific sepsis (explicit) (see Additional file 1: Table S1 for code extraction strategies) [2, 29]. ICU stays were retrieved from The Norwegian Intensive Registry (NIR) [25]. ICU patients were defined as any sepsis diagnosis and a registered ICU stay at the same admission. We used this strategy in the primary and up to 20 secondary co-existing ICD-10 discharge codes. Since the risk of recurrent sepsis admissions was higher at study start, we excluded all sepsis admissions between 2008 and 2010 and included index hospitalization from January 1, 2010, which coincided with available data from the Registry of the Norwegian National Social Security System.

We limited the study cohort to patients of working age (18–60 years), which is 2 years before the earliest retirement possibility in Norway. The rationale for the upper age limit was to identify patients who stopped working due to sepsis, as opposed to patients who retired unrelated to sepsis. We excluded patients with any disability pension prior to the sepsis hospitalization and patients who did not survive hospital discharge.

#### Definition of variables in the study

Working was defined by two criteria, and both had to be met. First, patients had to be registered with no sickness benefit or long-term medical benefit (work assessment allowance and permanent disability pension) for at least 90 of 121 days in the 6–2 months prior to sepsis admission to exclude those patients on sickness or medical benefits for other medical conditions than to sepsis as a cause of not being able to RTW. Second, patients had to be registered with a sickness benefit 31 days before the hospital admission date or 31 days after the hospital discharge date in order to identify those patients working before the sepsis admission.

For ICU patients, the cause of ICU admission was categorized as respiratory, circulatory, gastroenterological, neurological, sepsis, metabolic, renal, and other. Description of severity of disease was defined by Simplified Acute Physiology Score II (SAPS II) and number of patients receiving mechanical ventilation. COVID-19-related sepsis patients were included based on the presence of a code for COVID-19 (U07.1, U07.2) and ≥ one organ dysfunction code and/or explicit code. Infection sites and acute organ dysfunction were categorized by ICD-10 codes. ICD-10 discharge codes for selected comorbidities were based on diagnostic groups [30] (Additional file 1: Table S2). A readmission after hospitalization with sepsis was defined as an admission within 30 days after discharge, regardless of cause.

### Outcome measures

Main outcome was work status at 6 months, 1 year, and 2 years after discharge from index sepsis hospitalization. We categorized work status at each time point as RTW, ever RTW, never RTW, and dead. Patients without any sickness or medical benefit at the measurement point were categorized as RTW. Patients on sickness or medical benefit at all the measurement points were categorized as never RTW. Lastly, patients who had returned to work at an earlier point but were back on sickness or medical benefits were categorized as ever RTW. Secondary outcomes were trends in RTW during the whole study period. Further, we also investigated sustainable RTW, defined as the absence of any sickness or medical benefit for at least 31 consecutive days after discharge from sepsis hospitalization.

Death and death date were retrieved from the Norwegian Cause of Death Registry [31].

### Statistical analysis

Descriptive results are presented as frequencies with percent, means with standard deviation, and medians as appropriate.

Clinical characteristics of interest included sex, age-group (18–29, 30–39, 40–49, 50–60 years), number and type of comorbidities, site of infection, number and type of acute organ dysfunctions, ICU treatment, COVID-19-infection status, length of stay, cause of ICU admission, SAPS II and invasive ventilation and readmission within 30 days. Except for information on invasive ventilation and cause of ICU admission, there was no missing in the data. Participants without missing data on either of the two variables are shown as n (%). These descriptive analyses were also repeated in the patients that did not work before sepsis admission.

#### Main outcome

The Norwegian National Social Security System Registry contains information about all members’ entry and exit dates and degrees of sickness and medical benefits. To investigate the proportion of patients returning to work, we calculated sepsis survivors with status RTW, never RTW or dead at 6 months from discharge date. At 1 year and 2 years after discharge date, we additionally included the category ever RTW. All estimates were divided by all patients working prior to admission, subtracting those who died between each measure point. We also completed analyses stratified by treatment in the ICU vs non-ICU and by COVID-19-related vs non-COVID-19-related sepsis.

#### Secondary outcomes

To examine temporal trends in RTW, we calculated 6-month, 1-year, and 2-year RTW by calendar year. This was calculated as the proportions with RTW divided by the number of survivors after the index sepsis admission each year. To avoid potential bias of sepsis hospitalizations over time due to changing age distribution, the RTW proportion was standardized according to 10-year age-groups (18–29, 30–39, 40–49, 50–60 years) using the age distribution in 2011 as the base for non-ICU patients, and the age distribution in 2015 as the base for patients admitted to ICU. This was performed by the dstdize command in Stata using the option for individual-level data [32]. Temporal trends in age-standardized RTW were estimated from least-squares linear regression across calendar years and weighted by the inverse variance of the RTW proportion [33].

The probability of sustainable RTW were investigated using Cox regression to estimate crude and adjusted hazard ratios (HRs) with 95% confidence intervals (CIs). Association with age and sex was mutually adjusted, whereas all other associations were adjusted for both sex (male, female) and age (years). Comorbidities, site of infection, and acute organ dysfunctions were analyzed as categorical variables, using the most common category as the reference. These categories were mutually exclusive, and the analyses were conducted on a restricted sample of patients with none or only one infection site, comorbidity, or acute organ dysfunction, respectively. Two subgroup analyses of ICU vs. non-ICU patients and sepsis vs. COVID-19-related sepsis were conducted with similar approach as described above, except for entry date. The subgroup analysis of ICU vs. non-ICU patients was restricted to those sepsis patients that were hospitalized after May 1, 2014. We delayed start of follow-up for all since earlier information for the ICU patients was unavailable [25], and late entry for only one group may affect hazards between the groups. The analysis of sepsis vs COVID-19-related sepsis patients was conducted in the same way, and all patients were allowed entrance to the study only if they were hospitalized on or after February 28, 2020, corresponding to the first confirmed hospitalized COVID-19 case in Norway. In all Cox regression models, the patients were followed for 2 years after the date of discharge with an index sepsis admission to make sure the follow-up time covered the timespan of possible sick leave and was within the first possible retirement age. The discharge date was restricted to after July 1, 2010, to validate the sick-leave data and ensure the participants were in the workforce. In analysis of sustainable RTW, patients were censored at the date of sustainable RTW, death date, or the last day of follow-up (December 31, 2021). The last date for inclusion was October 1, 2021, to allow for a valid assessment of sustainable RTW. The proportional hazards assumption of the Cox model was examined by visual inspection of log–log plots.

As many individuals go on and off sickness benefits, we conducted a sensitivity analysis where sustainable RTW was defined as at least 92 consecutive days without any sickness benefit. Further, a sensitivity analysis was conducted including ICU and non-ICU patients. Since many patients have more than one infection site, comorbidity, and acute organ dysfunction, we also analyzed separate binary variables for each infection site, comorbidity, and acute organ dysfunction (i.e. respiratory infection site = 1, all other infection sites = 0).

All analyses were conducted using STATA version 16.1 (Stata Corp).

### Ethics

The study was approved by the Regional Committee for Medical and Health Research Ethics (REK) in Eastern Norway (2019/42772) and the Data Access Committee in Nord-Trøndelag Hospital Trust (2021/184). In accordance with the approval from the REK and the Norwegian law on medical research, the project did not require a written patient consent. This work was analyzed on TSD (Service for Sensitive Data) facilities, owned by the University of Oslo, operated, and developed by the TSD service group at the University of Oslo, IT Department (USIT).

## Results

**Fig. 1:**
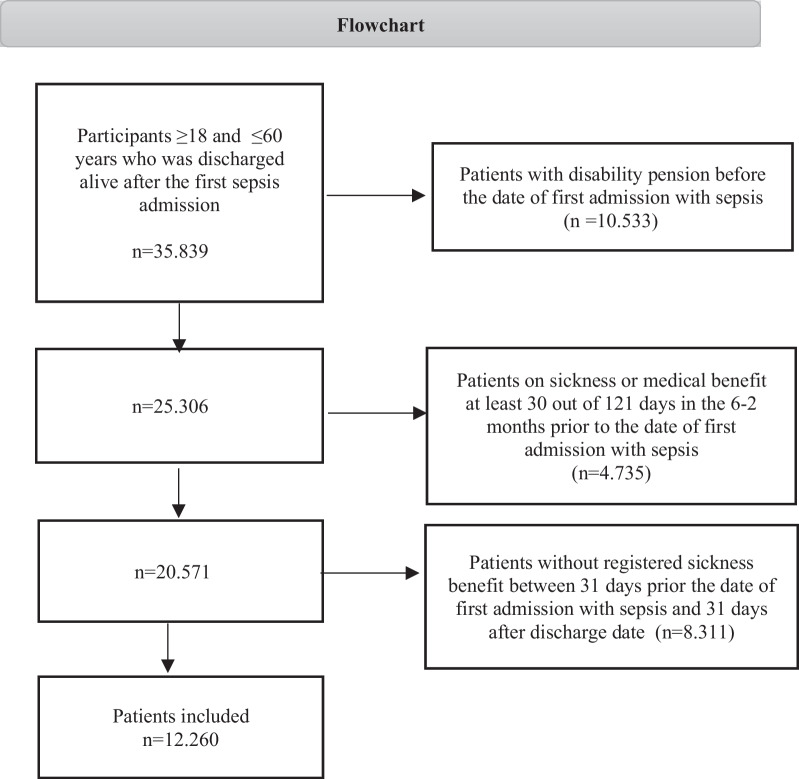
Flowchart of the selection process

Among 35.839 patients aged 18–60 years who were discharged alive from an index sepsis hospitalization during the study period, 12.260 (34.2%) were confirmed to be working prior to sepsis hospitalization and included in this study. 10.533 (29.3%) patients were excluded for disability pension prior to sepsis hospitalization, 4.735 (13.2%) patients were excluded for > 30 days of sickness or long-term medical benefits in the months prior to sepsis hospitalization indicating other illnesses than sepsis affecting RTW, and 8.311 (23.1%) patients were excluded for lack of registered sickness or medical benefit close to sepsis hospitalization. A flowchart of the exclusion and inclusion process is displayed in Fig. 1.

### Patient characteristics

Characteristics of the overall study cohort, including ICU and non-ICU patients, are shown in Table 1. Overall, the mean age was 43.7 years, and 59.9% were male. The number of sepsis patients admitted to the ICU was 951, accounting for 7.8% of the total study population with a mean age of 44.4 and an average SAPS II score of 33.4. Among ICU and non-ICU patients, the most common comorbidity was heart or vascular disease, as presented in 40.3% and 17.8%, respectively. The most common site of infection was respiratory, present in 52.4% of the ICU patients and 30.2% in the non-ICU patients. While respiratory acute organ dysfunction was the most common organ failure in ICU patients (51.0%), acute kidney dysfunction was the leading cause of organ failure among non-ICU patients (78.8%). The overall mean length of hospitalization was 13.9 days, accounting for 25.4 days in ICU patients and 12.9 days in non-ICU patients. Of all sepsis patients discharged alive, 29.9% were re-hospitalized within 30 days, and the ICU patients had a 47.8%. readmission rate.

**Table 1:** Characteristics of the 12.260 patients working prior to sepsis hospitalization, including ICU and non-ICU sepsis patients

	Sepsis, n (%)	Subgroups of sepsis	
		ICU, n (%)^a^	Non-ICU, n (%)^b^
Characteristics	12 260(100)	n = 951 (7.8)	n = 11 309 (92.2)
Male, n (%)	7 341 (59.9)	659 (69.3)	6 682 (59.1)
Age, years, mean (SD)	43.7 (11.8)	44.2 (11.8)	43.7 (11.8)
Age-group, n (%)			
18–29	2 077 (16.9)	155 (16.3)	1 922 (17.0)
30–39	2 314 (18.9)	179 (18.8)	2 135 (18.9)
40–49	3 121 (25.4)	222 (23.3)	2 899 (25.6)
50–60	4 748 (38.7)	395 (41.5)	4 353 (38.5)
Comorbidities, n (%)			
Heart and vascular	2 394 (19.5)	383 (40.3)	2 011 (17.8)
Cancer	1 941 (15.8)	44 (4.6)	1 897 (16.8)
Lung	681 (5.6)	71 (7.5)	610 (5.4)
Diabetes	666 (5.4)	81 (8.5)	586 (5.2)
Immune	269 (2.2)	10 (1.1)	259 (2.3)
Renal	145 (1.2)	8 (0.8)	137 (1.2)
Liver	44 (0.4)	7 (0.7)	37 (0.3)
Number of comorbidities, n (%)			
0	7 290 (58.5)	460 (48.4)	6 830 (60.4)
1	3 933 (32.1)	389 (40.9)	3 544 (31.3)
2	911 (7.4)	93 (9.8)	818 (7.2)
≥ 3	126 (1.0)	9 (1.0)	117 (1.0)
Site of infection, n (%)			
Respiratory	3 692 (30.2)	498 (52.4)	3 194 (28.2)
Genitourinary	1 602 (13.1)	87 (9.2)	1 515 (13.4)
Skin and soft tissue	558 (4.4)	43 (4.5)	515 (4.6)
Gastrointestinal	827 (6.8)	29 (3.1)	624 (5.2)
Intra-abdominal	755 (6.2)	59 (6.2)	696 (6.2)
Infections following a procedure	625 (5.1)	52 (5.5)	573 (5.1)
Endocarditis/myocarditis	190 (1.6)	14 (1.5)	176 (1.6)
Other^c^	2 056 (16.8)	210 (22.1)	1 846 (16.3)
COVID-19-related sepsis^d^	384 (3.1)	39 (4.1)	345 (3.1)
Organ system with acute dysfunction, n (%)			
Respiratory	3 063 (25.0)	486 (51.0)	2 578 (22.8)
Circulatory	878 (7.2)	243 (25.6)	635 (5.6)
Renal	2 627 (21.4)	230 (24.2)	2 397 (78.8)
Hepatic	194 (1.6)	31 (3.3)	163 (1.4)
Coagulation	757 (6.2)	36 (3.8)	721 (6.4)
Other^e^	2 543 (20.7)	293 (30.8)	2 250 (19.9)
Number of acute organ dysfunctions, n (%)			
1	6 422 (87.2)	559 (63.8)	5 675 (86.8)
2	736 (10.0)	224 (25.6)	656 (10.0)
3	164 (2.2)	67 (7.7)	146 (2.2)
≥ 4	42 (0.6)	26 (3.0)	62 (1.0)
Cause of ICU admission (n = 834), n (%)			
Respiratory	NA	173 (22.4)	NA
Circulatory	NA	117 (15.1)	NA
Gastroenterological	NA	38 (4.9)	NA
Neurological	NA	87 (11.2)	NA
Sepsis	NA	161 (20.8)	NA
Metabolic	NA	32 (4.1)	NA
Renal	NA	6 (0.8)	NA
Other	NA	220 (20.7)	NA
Illness severity at admission			
SAPS II, mean (SD) (n = 951)	NA	33.4 (17.3)	NA
Invasive ventilation (n = 585), n (%)	NA	485 (82.9)	NA
Length of hospital stay in days^f^, mean (SD)	13.9 (23.7)	25.4 (35.4)	12.9 (22.2)
30-day Readmission^g^, n (%)	3 664 (29.9)	456 (47.8)	3 208 (28.4)

Abbreviation: NA = Not Applicable. ICU = intensive care unit,, SD = standard deviation, SAPS II = Simplified Acute Physiology Score II

^a^Variable calculated from May 1, 2014

^b^Variable calculated from January 1, 2010

^c^Other infections = Bone, obstetric, upper airway, central nervous system and unknown

^d^Variable calculated from 28 February 2020

^e^Other acute organ dysfunction = Acidosis, unspecific gangrene, central nervous system dysfunctions and Systemic Inflammatory Response Syndrome

^f^Length of stay calculated as a total length of stay in hospital

^g^Readmission = admission within 30 days after discharge regardless of cause

### Main outcome

#### Return to work

In the total cohort, the proportion of RTW was 58.6% at 6 months, 67.5% at 1 year, and 63.4% at 2 years. Among patients admitted to ICU, the RTW proportion was 38.5% at 6 months, 53.6% at 1 year, and 52.2% at 2 years after discharge, while among non-ICU patients, the RTW proportion was 60.3% at 6 months, 68.6% at 1 year, and 64.2% at 2 years after discharge. In 2020 and 2021, for patients admitted with COVID-19-related sepsis, the RTW proportion was 66.9% and 77.8% at 6 months and 1 year after hospital discharge (Table 2).

**Table 2:** The proportion of RTW, never RTW and ever RTW and dead at 6 months, 1 year and 2 years in the period 2010 through 2021

Patient group and measurement point	n^a^	RTW^b^ (%)	Never RTW^c^ (%)	Ever RTW^d^ (%)	Dead (%)
All sepsis patients					
6 months	12 260	58.6	37.9	NA	3.4
1 year	11 751	67.5	21.6	5.6	5.4
2 years	10 845	63.4	15.3	13.7	7.7
Ward patients					
6 months	11 309	60.3	36.2	NA	3.4
1 year	10 856	68.6	20.4	5.6	5.4
2 years	10 085	64.2	14.4	13.7	7.6
ICU patients					
6 months	951	38.5	58.0	NA	3.5
1 year	895	53.6	36.2	4.8	5.4
2 years	760	52.2	26.7	13.0	8.0
COVID-19-related sepsis^e,f^					
6 months	384	66.9	32.6	NA	0.5
1 year	135	77.8	15.6	5.2	1.5

Abbreviation: RTW = return to work, NA = Not Applicable

^a^Includes all sepsis patients who survived an admission,

^b^Without medical benefit or disability pension at measurement point

^c^Patients at medical benefit or disability pension since discharge

^d^Patients without a medical benefit in a period after discharge, but back on medical benefit at measurement point

^e^Includes the years 2020 and 2021

^f^Includes patients admitted at ICU and non-ICU departments

### Secondary outcomes

#### Temporal trends in RTW

Overall, the annual age-standardized RTW proportion at 6 months was stable with a change of 0.14% (95% CI − 0.20 to 0.47), from 57.57% (95% CI 53.58 to 61.56) in 2010 to 63.10% (95% CI 58.23–67.87) in 2021. The annual age-standardized RTW proportion at 1 year also remained stable throughout the study period with a change of -0.45% (95% CI − 0.94 to 0.53), from 69.52% (95% CI 65.71–73.33) in 2010 to 64.89% (95% CI 61.56–68.22) in 2020. However, the 2-year age-standardized RTW declined by 1.51% (95% CI − 2.22 to − 0.79) per year over the study period, from 70.01% (95% CI 67.21–74.80) in 2010 to 57.04% (95% CI 53.81–60.28) in 2019.

**Fig. 2:**
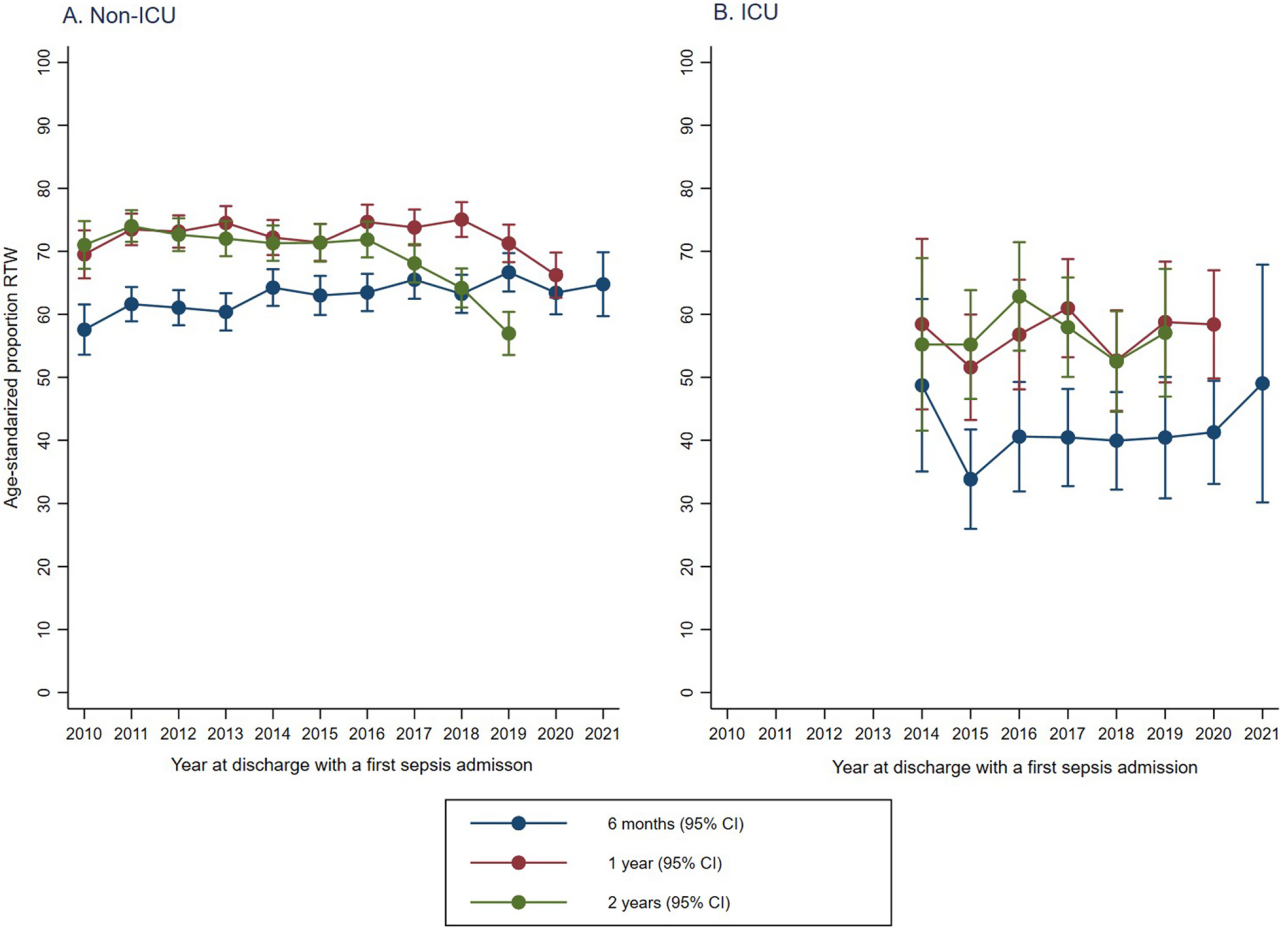
Age-standardized proportions RTW by discharge year for sepsis patients admitted **A**. Non-ICU (2010–2021) and **B**. ICU (2014–2021)

For patients admitted to the ICU, the annual age-standardized RTW proportion at 6 months, 1 year, and 2 years after discharge remained stable from 2014 through 2021, as shown in Fig. 2A and B and Additional file 1: Table S3. For non-ICU patients, the annual age-standardized RTW proportion at 6 months and 1 year after discharge remained stable throughout the study period. However, the 2-year age-standardized RTW declined by 1.32% (95% CI − 2.14 to − 0.49) per year over the study period, from 70.01% (95% CI 67.21–74.80) in 2010 to 56.96% (95% CI 53.54–60.38) in 2019. This decline was driven mainly by the years after 2016.

#### Characteristics associated with sustainable RTW

The median follow-up time for sustainable RTW was 0.2 years (range 0–2 years) and ended when a person started working. The results displayed in Table 3 show that patients and clinical characteristics were associated with sustainable RTW. Sepsis patients aged between 50 and 60 years had lower probability of sustainable RTW (HR 0.69; 95% CI 0.65–0.73) compared to younger sepsis patients (18–30 years). Sepsis patients with one comorbidity had an HR of 0.46 (95% CI 0.44–0.48) compared to no comorbidities, while sepsis patients with two acute organ dysfunctions had HR of 0.60 (0.55–0.66) compared to patients with one acute organ dysfunction. ICU patients had a 0.56 (95% CI 0.52–0.61) lower probability of sustained RTW compared to non-ICU patients. In addition, patients with genitourinary, gastrointestinal, and skin and soft tissue sites of infection had higher rates of sustainable RTW than the other infections sites, and COVID-19-related sepsis had a 1.31 (95% CI 1.15–1.49) higher chance of sustainable RTW compared to all sepsis patients.

**Table 3:** Associations of patient and clinical characteristics with sustained RTW

Variable	Person year (py) at risk	Events	Rate per py	Crude HR	Adjusted^a^ HR (95% C)
Age-group					
18–29	720	1 779	2.47	1.00	1.00 (Reference)
30–39	824	1 966	2.38	0.90	0.90 (0.84 − 0.96)
40–49	1 080	2 552	2.36	0.80	0.78 (0.75 − 0.85)
50–60	1 595	3 592	2.25	0.69	0.69 (0.65 − 0.73)
Sex					
Male	2 579	5 939	2.30	1.00	1.00 (Reference)
Female	1 639	3 950	2.41	1.03	1.01 (0.97 − 1.05)
ICU treatment^b^					
No	2 335	5 974	2.56	1.00	1.00 (Reference)
Yes	396	660	1.67	0.57	0.56 (0.52 − 0.61)
Sepsis subgroup^c^					
Sepsis	362	989	2.73	1.00	1.00 (Reference)
COVID-19-related	95	324	3.41	1.25	1.31 (1.15 − 1.49)
Site of infection^d^					
Respiratory	988	2 462	2.49	1.00	1.00 (Reference)
Genitourinary	260	785	3.01	1.40	1.38 (1.27 − 1.50)
Intra-abdominal	162	391	2.41	1.03	1.03 (0.92 − 1.14)
Gastrointestinal infections	169	637	3.80	1.69	1.64 (1.51 − 1.79)
Skin and soft tissue	105	276	2.64	1.26	1.27 (1.12 − 1.44)
Infections following a procedure	136	244	1.80	0.83	0.84 (0.73 − 0.96)
Endocarditis/myocarditis	46	74	1.60	0.70	0.70 (0.56 − 0.88)
Other infections^e^	793	1 800	2.27	1.00	0.96 (0.91 − 1.02)
Comorbidities^d^					
Heart and vascular	652	1 175	1.80	1.00	1.00 (Reference)
Cancer	749	736	0.98	0.52	0.52 (0.48 − 0.58)
Lung	139	367	2.65	1.57	1.60 (1.42 − 1.80)
Diabetes	97	269	2.80	1.58	1.58 (1.39 − 1.81)
Renal	17	25	1.48	0.99	0.98 (0.66 − 1.46)
Immune	29	99	3.42	2.07	2.06 (1.67 − 2.53)
Liver	5	10	2.03	0.60	0.62 (0.33 − 1.15)
No. of comorbidities					
0	2 113	6 598	3.12	1.00	1.00 (Reference)
1	1 687	2 681	1.59	0.45	0.46 (0.44 − 0.48)
2	377	547	1.45	0.38	0.39 (0.36 − 0.42)
≥ 3	41	63	1.55	0.30	0.32 (0.25 − 0.41)
Type of acute organ dysfunction^d^					
Respiratory	819	1 858	2.27	1.00	1.00 (Reference)
Renal	563	1 761	3.12	1.45	1.48 (1.39 − 1.58)
Circulatory	187	353	1.89	0.89	0.91 (0.82 − 1.03)
Coagulation	332	407	1.23	0.62	0.64 (0.58 − 0.71)
Hepatic	37	66	1.79	0.83	0.82 (0.64 − 1.04)
Other acute organ dysfunctions^f^	275	735	2.67	1.33	1.30 (1.19 − 1.41)
No. of acute organ dysfunctions					
1	2 214	5 180	2.34	1.00	1.00 (Reference)
2	337	492	1.46	0.60	0.60 (0.55 − 0.66)
3	82	109	1.33	0.57	0.56 (0.46 − 0.68)
≥ 4	18	25	1.42	0.49	0.49 (0.33 − 0.72)

Abbrevation: HR =hazard ratio, CI = confidence interval, ICU = intensive care unit,

^a^Cox regression with time to death as dependent variable, the listed variable as covariate (one at the time), and sex and age

^b^Enter date = May 1, 2014

^c^Enter date = February 28, 2020

^d^Categorical variable where one ICD-10 code excludes other ICD-10 codes in the same diagnosis group

^e^Other infections = Bone, obstetric, upper airway, central nervous system and unknown

^f^Other acute organ dysfunctions = Acidosis, unspecific gangrene, central nervous system dysfunctions and Systemic Inflammatory Response Syndrome

### Sensitivity analysis

A sensitivity analysis of the 8.311 individuals who were excluded from this study due to no evidence of work prior to the sepsis episode was conducted. Compared to patients working before sepsis hospitalization, these patients were younger (mean age 40.5 (SD = 13.2) vs. 43.7 years, SD = 11.8) and consisted of more women (46.2% vs. 40.1%) (Additional file 1: Table S4).

In the sensitivity analyses of ICU and non-ICU patients investigating sustained RTW and changing the definition of sustainable RTW to working at least 92 consecutive days after discharge date with an index sepsis episode, the adjusted hazard ratio from Cox regression did not differ from the results in Table 3, see Additional file 1: Table S5 and Table S6. Sensitivity analysis with binary categories is presented in Additional file 1: Table S7, confirms our analyses and estimates with mutually exclusive categories.

## Discussion

Our study is the first to use complete nationwide registries to estimate RTW in sepsis patients, and we demonstrate that RTW is a challenge for many patients, even 2 years after discharge. Our estimates show that a higher proportion of patients with sepsis returned to work at 1 year compared to 6 months and 2 years after discharge. The trends in RTW were stable throughout the study period, except for non-ICU patients where we observed a yearly decrease in RTW at 2 years after hospitalization. Further, we found that decreasing age, fewer comorbidities, and fewer acute organ dysfunctions were associated with sustainable RTW in sepsis survivors. In addition, we found that ICU sepsis patients had a lower probability of sustainable RTW than non-ICU sepsis patients. COVID-19-related sepsis patients had a higher probability of achieving sustainable RTW than ICU sepsis patients and sepsis patients without COVID-19.

Previous studies have found that ICU patients self-reported RTW at 1 year between 55% and 78% [34]. The only previous registry-based study found that 60% of the ICU patients, regardless of diagnosis, returned to work at 1 year [9]. For comparison, we found that 53.6% of the ICU patients with sepsis had returned to work at 1 year. Previous studies have found worse overall functioning outcomes in sepsis survivors compared to other intensive care survivors [11, 12], and since we only included sepsis patients, our result in the lower range is expected.

A systematic review of ICU studies suggests increasing rates of RTW over time [34], but the included studies have a high degree of RTW variability [9, 34–39]. While we use a national mandatory registry to calculate RTW, 51 of the 52 included studies in this systematic review used self-reported answers to questions in face-to-face or telephone interviews or mailed questionnaires to collect data. The majority of the included studies had more than 10% lost to follow-up since ICU/hospital discharge [34], while our complete national registries including weekly updates on RTW enabled us to follow-up everybody until death date and thus no loss to follow-up.

Investigating sepsis and non-sepsis ICU patients, organ support therapy and RTW, Riddersholm et al. [9] found that 29% were back on medical benefits within 1 year. For comparison, this is higher than our result (4.8%) at 1 year among sepsis patients admitted to ICU. If we compare the RTW proportion at 2 years, Riddersholm et al. found that 68% of the patients returned to work, which is higher than our age-standardized proportion at 2 years (58.4%). As previously argued, sepsis patients are expected to have worse outcomes than other critically ill patients, thus the diverging result. In addition, there are differences in social infrastructure with earlier transfer to long-term medical benefit in Denmark than in Norway [28, 40]. Thus, potential differences may not all be health-related but may also be influenced by social infrastructure.

The fact that the ICU patients’ proportion of RTW was stable can be explained by stable mortality in patients receiving ICU treatment over time in the cohort [41, 42], i.e., the same proportion of ill ICU sepsis patients survive and are potential candidates for RTW. Interestingly, we found a decreasing trend of 2-year RTW for non-ICU sepsis patients from 2010 through 2021. To our knowledge, trends in RTW in sepsis patients are not previously described. Based on our previous works, we observed decreasing case fatality rates and decreasing 1- and 2-year mortality in non-ICU patients in this population [41, 42], pointing to a higher proportion of sepsis survivors with possible physical and cognitive sequela. We therefore hypothesize that the decreasing trend in RTW can be explained by this increased survival.

To our knowledge, one previous study has used administrative data covering 30% of the German population to estimate RTW in sepsis patients in general with a follow-up to 12 months after discharge [13]. They found that 69% returned to work at 6 months and 76% returned to work at 12 months. This is a much higher RTW rate than in our study. While they extracted explicit sepsis ICD-10 codes, we extracted both implicit and explicit ICD-10 sepsis codes. Notably, an explicit approach has previously been found to underestimate sepsis estimates and may be an explanation of the diverging results [43].

A Danish study of RTW in COVID-19 patients found that 6.6% of the patients hospitalized with COVID-19 did not work at 3 months [44]. In contrast, we found that 33.1% of patients with COVID-19-related sepsis were not working at 6 months. Both our and the Danish study (2022) were based on registry data. However, while the Danish study included all hospitalized patients with a positive SARS-CoV-2 polymerase chain reaction (PCR) test, regardless of organ dysfunction, our study focused on all COVID-19 patients with acute organ dysfunctions, regardless of COVID-19 as main or secondary diagnosis. This difference in patient selection with more severely sick patients in our study may explain the diverging results.

Associations between patients and clinical characteristics and RTW in sepsis patients are limited. A recent registry-based Danish study (2018) of ICU patients and the need for organ support therapy found mechanical ventilation to be associated with a decreased chance of RTW [9]. Another study investigating different severity stages of acute kidney injury (AKI, stages I-III) in ICU patients surviving acute respiratory failure and/or sepsis found that 50% of those with AKI I and 22% of those with AKI II-III returned to work at 3-month follow-up [45]. Our study found that acute renal dysfunctions had a higher probability of sustainable RTW compared to acute respiratory dysfunction. However, we did not have the availability of AKI stages to differentiate and compare directly to Riddersholm et al. Another study by Poulsen et al. [49] investigating patients with septic shock found that 43% of patients returned to work at 1 year [46]. This is lower than in our study, where approximately 58% of the ICU patients RTW at 1 year. The diverging result may be explained by differences in the severity of sepsis since our study included all patients with sepsis receiving ICU treatment and not only septic shock. To our knowledge, no previous study has investigated characteristics associated with the probability of sustainable RTW in a patient group consisting of only patients admitted with sepsis, including non-ICU sepsis patients. Our findings support results from previous studies reporting that increasing age and pre-existing chronic comorbidities are associated with work status [35, 36]. However, compared to our study, these studies were small and based on self-reports; thus, direct comparisons are difficult.

There are some limitations to our study. First, the sepsis cohort is extracted from NPR using ICD-10 codes. We used the Sepsis-3 definition throughout the study period when extracting ICD-10 codes, albeit the definition first came in 2016 [1]. Second, implicit sepsis codes are found to overestimate sepsis while explicit sepsis codes often underestimate sepsis, and by using both approaches, we assume to align the chances for over- and underestimation [43]. Moreover, we cannot be sure that all acute organ dysfunction codes are associated with the infection, and thus, this could generate an overestimation of sepsis. Third, we identified only 34.2% as working, which is much lower than the national employment rate of 80.2%. Identifying a low employment rate among sepsis survivors may underestimate RTW. In our study, this could be a result of the strict inclusion criteria where only patients on medical benefit for a sepsis episode was considered. The self-employed are only entitled to sickness benefits after the 17th day and therefore may have returned to work without a registered sick leave. Fourth, we did not investigate whether patients received graded sickness or medical benefits, meaning that some could have partly returned to work. Notably, the incidence rate and case fatality are in the lower range compared to estimates from a recent meta-analysis from 2020 and the global burden of disease study from 2017 [2, 41, 47]. The RTW estimates may also be influenced by social infrastructure, and therefore, the interpretation of the analysis is primarily relevant to countries with the same burden and comparable social welfare systems. Fifth, the low percentage of ICU patients can be explained by inclusion of only working patients, where the majority were without comorbidities; thus, the risk of complicated organ dysfunction and a need of ICU stay was lowered. However, the low percentage can also be explained by how the Norwegian hospitals are organized in ICU, intermediate care wards and wards [48]. Intermediate care awards provides noninvasive ventilation and vasopressor support and does not report to NIR; thus, we were unable to determine the percentage of sepsis patients in need of organ support therapy. Since only SAPS II score is mandatory to report to NIR, we could not report on SOFA score. Sixth, we found a high prevalence of cancer patients in the overall cohort. This may be a result of a broad comorbidity extraction strategy of ICD-10 cancer codes. However, there is well known that cancer patients have an increased risk of developing sepsis [49], and the higher prevalence among non-ICU sepsis patients than ICU sepsis patients suggests that the majority are managed at wards. Lastly, by using mutually exclusive categories of infection site, acute organs dysfunction and comorbidities to assess sustainable RTW we risk oversimplifying a more complex issue and ignoring important nuances. However, sensitivity analysis with binary variables identified the same infection sites, comorbidities, and acute organ dysfunctions as the main analysis, and therefore, we consider our estimates representative.

One major strength is that our study is based on complete national administrative data [50]; thus, the selection bias is minimized. We have complete follow-up data, which is not possible in other cohorts based on patients’ self-report, where over 10% lost to follow-up since discharge is common [34]. We studied characteristics associated with sustainable RTW, while previous studies report only RTW at fixed time points after receiving ICU treatment. Our group of patients was defined as RTW if they were without any form of benefits and had sustained work for at least 31 consecutive days; thus, our results account for the fact that they have probably resumed work after the index sepsis episode. This is a strength because estimates of only fixed time points are snapshots of RTW and lack sustainable RTW. Furthermore, another strength is that we studied RTW over 11 years, thus making it possible to detect RTW trends and report RTW results beyond 1 year. Finally, the criteria for sickness benefits and disability pension have been the same during the study period reducing the chance that changes in RTW reflect changes in criteria for medical benefits.

Dealing with sepsis can result in long hospital stays, and it can take time returning to work. In cancer patients, research suggests that multidisciplinary interventions involving physical, psychoeducational, and/or vocational components lead to more patients returning to work compared to usual care [51]. The literature is scare regarding effects of rehabilitation of patients with sepsis [52]; however, a systematic review of cancer patients found that while some interventions showed a significant difference for work outcomes when compared to usual care, others did not [53]. In conclusion, while work facilitation efforts may have a potential to increase RTW rates, more research is needed to identify the most effective strategies and interventions among sepsis patients.

## Conclusion

Half of the ICU sepsis patients and two-third of the non-ICU sepsis patients had resumed work at 2 years. There were no improvements in RTW proportions over time. Vulnerable groups with reduced probability of sustainable RTW were patients at higher ages, patients with an increasing number of comorbidities, and patients with an increasing number of acute organ dysfunctions. The lack of progress in improving RTW in patients with sepsis should warrant targeted interventions to improve long-term outcomes.

### Supplementary Information

**Additional file 1.** Supplementary figure and tables.

## Data Availability

No additional data available. We do not have ethical approval to deposit our datasets in publicly available repositories. Researchers need approval by the Regional Ethical Committee for handling of NPR, NIR and NAV data files. NPR has precise information on all data exported to different projects and there are no restrictions regarding data export given REK approval.

## References

[1] Singer M, Deutschman CS, Seymour CW, Shankar-Hari M, Annane D, Bauer M (2016). The third international consensus definitions for sepsis and septic shock (Sepsis-3). JAMA.

[2] Rudd KE, Johnson SC, Agesa KM, Shackelford KA, Tsoi D, Kievlan DR (2020). Global, regional, and national sepsis incidence and mortality, 1990–2017: analysis for the global burden of disease study. Lancet.

[3] Prescott HC, Iwashyna TJ, Blackwood B, Calandra T, Chlan LL, Choong K (2019). Understanding and enhancing sepsis survivorship. Priorities for research and practice. Am J Respir Crit Care Med.

[4] Iwashyna TJ, Ely EW, Smith DM, Langa KM (2010). Long-term cognitive impairment and functional disability among survivors of severe sepsis. JAMA.

[5] Uleberg O, Pape K, Kristiansen T, Romundstad PR, Klepstad P (2019). Population-based analysis of the impact of trauma on longer-term functional outcomes. Br J Surg.

[6] Ardolino A, Sleat G, Willett K (2012). Outcome measurements in major trauma–results of a consensus meeting. Injury.

[7] Jones C, Griffiths RD (2013). Mental and physical disability after sepsis. Minerva Anestesiol.

[8] Rothenhausler HB, Ehrentraut S, Stoll C, Schelling G, Kapfhammer HP (2001). The relationship between cognitive performance and employment and health status in long-term survivors of the acute respiratory distress syndrome: results of an exploratory study. Gen Hosp Psychiatry.

[9] Riddersholm S, Kragholm K, Rasmussen BS, Christensen S, Christiansen CF. Organ failure and return to work after intensive care. Critical Care. 2018;22. 10.1007/s00134-018-5157-1 29616288

[10] McPeake J, Mikkelsen ME, Quasim T, Hibbert E, Cannon P, Shaw M (2019). Return to employment after critical illness and its association with psychosocial outcomes. A systematic review and meta-analysis. Ann Am Thorac Soc.

[11] Kayambu G, Boots RJ, Paratz JD (2011). Early rehabilitation in sepsis: a prospective randomised controlled trial investigating functional and physiological outcomes The i-PERFORM Trial (Protocol Article). BMC Anesthesiol.

[12] Hayes JA, Black NA, Jenkinson C, Young JD, Rowan KM, Daly K (2000). Outcome measures for adult critical care: a systematic review. Health Technol Assess.

[13] Fleischmann-Struzek C, Ditscheid B, Rose N, Spoden M, Wedekind L, Schlattmann P (2023). Return to work after sepsis-a German population-based health claims study. Front Med (Lausanne).

[14] Soriano JB, Murthy S, Marshall JC, Relan P, Diaz JV (2022). A clinical case definition of post-COVID-19 condition by a Delphi consensus. Lancet Infect Dis.

[15] Fernández-de-Las-Peñas C (2022). Long COVID: current definition. Infection.

[16] Akbarialiabad H, Taghrir MH, Abdollahi A, Ghahramani N, Kumar M, Paydar S (2021). Long COVID, a comprehensive systematic scoping review. Infection.

[17] PASC Daschboard https://pascdashboard.aapmr.org/

[18] Hodgson CL, Higgins AM, Bailey MJ, Mather AM, Beach L, Bellomo R (2021). The impact of COVID-19 critical illness on new disability, functional outcomes and return to work at 6 months: a prospective cohort study. Crit Care.

[19] Lindahl A, Aro M, Reijula J, Mäkelä MJ, Ollgren J, Puolanne M (2022). Women report more symptoms and impaired quality of life: a survey of Finnish COVID-19 survivors. Infect Dis (Lond).

[20] Carenzo L, Dalla Corte F, Haines RW, Palandri C, Milani A, Aghemo A (2021). Return to work after Coronavirus disease 2019 acute respiratory distress syndrome and intensive care admission: prospective, case series at 6 months from hospital discharge. Crit Care Med.

[21] van Veenendaal N, van der Meulen IC, Onrust M, Paans W, Dieperink W, van der Voort PHJ (2021). Six-month outcomes in COVID-19 ICU patients and their family members: a prospective cohort study. Healthcare (Basel)..

[22] Garrigues E, Janvier P, Kherabi Y, Le Bot A, Hamon A, Gouze H (2020). Post-discharge persistent symptoms and health-related quality of life after hospitalization for COVID-19. J Infect.

[23] Garzillo EM, Cioffi A, Carta A, Monaco MGL (2022). Returning to Work after the COVID-19 pandemic earthquake: a systematic review. Int J Environ Res Public Health.

[24] Bakken IJ, Ariansen AMS, Knudsen GP, Johansen KI, Vollset SE (2020). The Norwegian patient registry and the Norwegian registry for primary health care: research potential of two nationwide health-care registries. Scand J Public Health.

[25] Norwegian Intensive Registry https://helse-bergen.no/norsk-intensivregister-nir

[26] Statistics Norway https://www.ssb.no/en

[27] NAV. Membership of the National Insurance Scheme 2019 https://www.nav.no/en/home/rules-and-regulations/membership-of-the-national-insurance-scheme

[28] NAV 2023 https://www.nav.no/en/home

[29] Angus DC, Linde-Zwirble WT, Lidicker J, Clermont G, Carcillo J, Pinsky MR (2001). Epidemiology of severe sepsis in the United States: analysis of incidence, outcome, and associated costs of care. Crit Care Med.

[30] Stausberg J, Hagn S (2015). New morbidity and comorbidity scores based on the structure of the ICD-10. PLoS ONE.

[31] The norwegian cause of death registry 2014 https://www.fhi.no/globalassets/dokumenterfiler/helseregistre/dar/dodelighet-og-dodsarsaker-pdf.pdf

[32] STATA. Dstdize: Direct and indirect standardization: Stata.com; 2014 https://www.stata.com/manuals13/rdstdize.pdf

[33] Kim HJ, Fay MP, Feuer EJ, Midthune DN (2000). Permutation tests for joinpoint regression with applications to cancer rates. Stat Med.

[34] Kamdar BB, Suri R, Suchyta MR, Digrande KF, Sherwood KD, Colantuoni E (2020). Return to work after critical illness: a systematic review and meta-analysis. Thorax.

[35] Kamdar BB, Huang M, Dinglas VD, Colantuoni E, Von Wachter TM, Hopkins RO (2017). Joblessness and lost earnings after acute respiratory distress syndrome in a 1-year national multicenter study. Am J Respir Crit Care Med.

[36] Oeyen S, Vandijck D, Benoit D, Decruyenaere J, Annemans L, Hoste E (2007). Long-term outcome after acute kidney injury in critically-ill patients. Acta Clin Belg.

[37] von Bahr V, Kalzen H, Hultman J, Frisen KG, Dobrosavljevic T, Holzgraefe B (2019). Long-term pulmonary function and quality of life in adults after extracorporeal membrane oxygenation for respiratory failure. Perfusion (United Kingdom).

[38] Myhren H, Ekeberg Ø, Stokland O (2010). Health-related quality of life and return to work after critical illness in general intensive care unit patients: a 1-year follow-up study. Crit Care Med.

[39] Eddleston JM, White P, Guthrie E (2000). Survival, morbidity, and quality of life after discharge from intensive care. Crit Care Med.

[40] Resource flow performance during resource flows: borger.dk; 2023 [https://www.borger.dk/arbejde-dagpenge-ferie/fleksjob-loentilskud-for-foertidspensionister-revalidering/ressourceforloebsydelse-under-ressourceforloeb.

[41] Skei NV, Nilsen TIL, Knoop ST, Prescott H, Lydersen S, Mohus RM (2023). Long-term temporal trends in incidence rate and case fatality of sepsis and COVID-19-related sepsis in Norwegian hospitals, 2008–2021: a nationwide registry study. BMJ Open.

[42] Skei NV, Nilsen TIL, Mohus RM, Prescott HC, Lydersen S, Solligård E, et al. Trends in mortality after a sepsis hospitalization: a nationwide prospective registry study from 2008 to 2021. Infection. 2023. 10.1007/s15010-023-02082-z PMC10665235 37572240

[43] Fleischmann-Struzek C, Thomas-Ruddel DO, Schettler A, Schwarzkopf D, Stacke A, Seymour CW (2018). Comparing the validity of different ICD coding abstraction strategies for sepsis case identification in German claims data. PLoS ONE.

[44] Jacobsen PA, Andersen MP, Gislason G, Phelps M, Butt JH, Køber L (2022). Return to work after COVID-19 infection: A Danish nationwide registry study. Public Health.

[45] Mayer KP, Ortiz-Soriano VM, Kalantar A, Lambert J, Morris PE, Neyra JA (2022). Acute kidney injury contributes to worse physical and quality of life outcomes in survivors of critical illness. BMC Nephrol.

[46] Poulsen JB, Møller K, Kehlet H, Perner A (2009). Long-term physical outcome in patients with septic shock. Acta Anaesthesiol Scand.

[47] Fleischmann-Struzek C, Mellhammar L, Rose N, Cassini A, Rudd KE, Schlattmann P (2020). Incidence and mortality of hospital- and ICU-treated sepsis: results from an updated and expanded systematic review and meta-analysis. Intensive Care Med.

[48] Bertnum AB, Fragapane GI, Semini M, Strandhagen JO, editors. Possibilities and benefits of intermediate care units in healthcare systems from a logistics perspective. Advances in production management systems production management for data-driven, intelligent, collaborative, and sustainable manufacturing; 2018 2018; Cham: Springer International Publishing.

[49] Danai PA, Moss M, Mannino DM, Martin GS (2006). The epidemiology of sepsis in patients with malignancy. Chest.

[50] Aasdahl L, Fimland MS, Bjørnelv GMW, Gismervik S, Johnsen R, Vasseljen O, et al. Economic evaluation of inpatient multimodal occupational rehabilitation vs. outpatient acceptance and commitment therapy for sick-listed workers with musculoskeletal- or common mental disorders. J Occup Rehabil. 2023. 10.1007/s10926-022-10085-0 PMC10495483 36949254

[51] de Boer AGEMTT, Tamminga SJ, Feuerstein M, Frings-Dresen MHW, Verbeek JH (2015). Interventions to enhance return-to-work for cancer patients. Cochrane Database Syst Rev.

[52] Taito S, Taito M, Banno M, Tsujimoto H, Kataoka Y, Tsujimoto Y (2018). Rehabilitation for patients with sepsis: a systematic review and meta-analysis. PLoS ONE.

[53] Algeo N, Bennett K, Connolly D (2021). Rehabilitation interventions to support return to work for women with breast cancer: a systematic review and meta-analysis. BMC Cancer.

