# Supplementary material for: Return to work after hospitalization for sepsis: a nationwide, registry-based cohort study
Source: Crit Care. 2023 Nov 15;27:443. doi: 10.1186/s13054-023-04737-7 (PMC10652599; doi:10.1186/s13054-023-04737-7)
Supplement: Supplementary file 1 — Additional file 1. Supplementary figure and tables. [file 13054_2023_4737_MOESM1_ESM.docx]

# Additional File 1

Contents

[**Supplementary File 1 :** Data sources 2](#_Toc150890408)

[The Norwegian Patient Registry 2](#_Toc150890409)

[The Norwegian Intensive Registry 2](#_Toc150890410)

[The Norwegian National Social Security System Registry 2](#_Toc150890411)

[The Norwegian Cause of Death Registry 3](#_Toc150890412)

[**Supplementary Table 1** Overview of ICD-10 codes identifying explicit and implicit sepsis^6^ 4](#_Toc150890413)

[**Supplementary Table 2** Overview of ICD-10 codes identifying comorbidities and categories of infection sites and acute organ dysfunctions 5](#_Toc150890414)

[**Supplementary Table 3** Age-standardized RTW among patients discharged from ICU (n=951) 6](#_Toc150890415)

[**Supplementary Table 4** Characteristics of patients working and patients without medical benefit prior sepsis admission and discharged alive 7](#_Toc150890416)

[**Supplementary Table 5** Associations of patient and clinical characteristics with sustained RTW in ICU and non-ICU sepsis patients 8](#_Toc150890417)

[**Supplementary Table 6** Adjusted hazard ratio from Cox regression by characteristics of patients working in at least 92 consecutive days without sickness benefit after index sepsis admission. 10](#_Toc150890418)

[**Supplementary Table 7** Adjusted hazard ratio from Cox regression by characteristics of patients working in at least 31 consecutive days without sickness benefit after index sepsis admission (not mutually exclusive categories) 11](#_Toc150890419)

[References 12](#_Toc150890420)

# **Supplementary File 1 :** Data sources

### The Norwegian Patient Registry

The Norwegian Patient Registry (NPR) was established by a research institute (SINTEF) in 1997 and transferred to the Norwegian Directorate of Health in 2007. Before the transfer the data did not include PIN, but from 2008 reporting with PIN is mandatory ^1^. The registry covers all public specialist health-care services in Norway, including private institutions and medical specialists contracted to the regional health authorities. The coded medical information is classified according to ICD-10 diagnostic codes in one primary diagnosis and up to twenty secondary diagnoses ^2^. Reporting to the NPR is mandatory for all public Norwegian hospitals.

### The Norwegian Intensive Registry

The Norwegian Intensive Care Registry (NIR) covers all intensive care unit (ICU) admissions, and data are available from 1 May 2014 ^3^. NIR contains information on all patients treated at intensive care units in Norway, including patients admitted with COVID-19. NIR collects individual data from all ICU‐admissions in Norway, recorded securely via a web‐based platform. Inclusion criteria in the NIR includes one of the following 5 criteria :

1. Length of stay over 24 hours in intensive care.

2. Require invasive mechanical ventilation.

3. Are transferred between intensive care wards.

4. Persistent administration of vasoactive medication

5. Passed away during stay in intensive care regardless of length of stay.

### The Norwegian National Social Security System Registry

The Norwegian Work and Welfare Administration (NAV) runs the Norwegian National Social Security System Registry and operates all social benefits, which all Norwegian citizens have access to through a compulsory membership in The Norwegian National Insurance Scheme ^4^. The Norwegian National Social Security System Registry contains information about all members` sickness and medical benefits including entry and exit dates for the benefit received and degrees thereof . Medical benefit during illness is managed by medical doctors that need to send a sick leave application on behalf of the patient to NAV. All current medical benefits, including medical benefits, work assessments allowance and permanent disability pensions, are available from 2010.

We used data from NAV containing start and stop dates and degree and type of medical benefit, work assessments allowance and disability pension.

### The Norwegian Cause of Death Registry

The Norwegian Cause of Death Registry (DÅR) is mandatory and **contains information on deaths and causes of death in Norway from 1951 until today** and collects data on deaths by age, sex, cause, place of death, and place of residence for Norway ^5^. The DÅR is managed by the Norwegian Institute of Public Health (NIPH). The registry collects death certificates for all deaths that occur in the country. It also registers the deaths of Norwegians who die abroad.

| **Supplementary Table 1** Overview of ICD-10 codes identifying explicit and implicit sepsis^6^ | |
| --- | --- |
| Sepsis, Explicit code strategy | A02.1, A20.7, A21.7, A22.7, A24.1, A26.7, A28.2, A32.7, A39.2, A39.4, A40, A41, A42.7, B00.7, B37.7 |
| Sepsis^a,b^ Implicit code strategy | **Infection**  A00/09, A19/28, A30/32, A36/39, A42/44, A46, A48/49, A54, A59, A69.0, A69.1, A69.9, A70,  A74/75, A77/81, A83/89, A92/99,  B00/09, B25/27, B33/34, B37/46, B48/50, B54/55, B57/58, B60, B64, B67, B95/97, B99,  G00/08,  H05.0, H60.2, H70.0,  I00, I33, I38/40.0,  J01/06, J09/22, J36, J39.0, J39.1, J85, J86,  K35/37, K61, K63.0/63.1, K65, K75.0, K81.0, K83.0, L02/04, L08,  M00/01, M72.6, M86,  N10, N15.1, N30, N39.0, N41.0, N41.2, N41.3, N45, N70/74, N98.0, N49,  O03.0, O03.5, O04.5, O08.0, O23, O75.3, O85/86, O88.3, O91, O98,  T80.2, T81.4, T82.6/82.7, T83.5/83.6, T84.5/84.7, T85.7, T88.0, U04, U07.1, U07.2 |
|  | AND |
|  | **Acute organ dysfunction**  D65, D69.5, E87.2, G93.4, I46, I95.9, J80, J95.2, J96, K72.0, K72.9, N00, N17, N99.0, R02, R09.0, R09.2, R40.0/40.2, R41, R55, R57, R57.2, R65.1 |
| Abbreviation: ICD= International Classification of Diseases  ^a^ Implicit sepsis was defined if one code of infection was present with at least one acute organ dysfunction within same hospital entry. Total sepsis estimates are calculated from both explicit and implicit cases.  ^b^ Explicit codes are excluded from infection codes | |

| **Supplementary Table 2** Overview of ICD-10 codes identifying comorbidities and categories of infection sites and acute organ dysfunctions | |
| --- | --- |
| **Comorbidities** | **ICD-10** code |
| Chronic heart- and vascular disease | G45, H34, I00/31, I34/37, I42/45, I47/95.8, I97/99 |
| Cancer | C00/97, D32/33, D35.2/35.4, D42, D43, D44.3/44.5, D45/47 |
| Chronic lung disease | J41/47, J84, J98 |
| Chronic renal disease | N18.3/18.5 |
| Diabetes | E10/11 |
| Dementia | F00/03, G30, G31.0, G31.2, G31.8 |
| Chronic immune disease | D80/84, Z94.0/94.4, Z94.8 |
| Chronic liver disease | K70.4, K72 |
| **Infection sites^a^** |  |
| Respiratory | J09/18, J20/22, J85/86, U07.1, U07.2 |
| Genitourinary | N10, N15.1, N30, N39.0, N41.0, N41.2/41.3, N45, N49, N70, N71/74, N98.0 |
| Gastrointestinal | A00/09 |
| Intra-abdominal | K35/37, K57, K61/61.1 K61.3, K63.0/63.1, K65, K75.0, K81.0, K83.0 |
| Endocarditis/myocarditis | I32/33, I38/41 |
| Skin/ Soft tissue | A46, B08/09, L02/04, L08, M72.6 |
| Infection after procedure | T80.2, T81.4, T82.6/82.7, T83.5/83.6, T84.5/84.7, T85.7, T88 |
| Other | A19/28, A30/32, A36/39, A42/44, A48/49, A54, A59, A69.0, A69.1, A69.9, A70, A74/75, A77/80, A81, A83/89, A92/B06, B25/27, B33/34, B37/46, B48/50, B54/55, B57/58, B60, B64, B67, B95/97, B99, G00/08, H05.0, H60.2, H70.0, J01/06, J36, J39.0/39.1, M00/01, M86, O03.0, O03.5, O04.5, O08.0, O23, O75.3, O85/86, O88.3, O91, O98 |
| **Acute organ dysfunction** |  |
| Respiratory | J80, J95.2, J96, R09.0, R09.2 |
| Circulatory | I46, I95.9, R57, R57.2 |
| Renal | N00, N17, N99.0 |
| Hepatic | K72.0, K72.9 |
| Coagulation | D65, D69.5 |
| Other acute organ dysfunctions | G93.4, R40.0/40.2, R41, R55, E87.2, R02, R65.1^b^ |
| ^a^ Explicit codes are excluded from other infection sites.  ^b^ R65.1 was excluded in the count of acute organ dysfunctions if present in combination with R57.2, according to the Norwegian ICD-10 coding rules. | |

| **Supplementary Table 3** Age-standardized RTW among patients discharged from ICU (n=951) | | | | | | | | | |
| --- | --- | --- | --- | --- | --- | --- | --- | --- | --- |
| 6 months | | | | 1 year | | | 2 years | | |
| year | n | crude | adjusted | n | crude | adjusted | n | crude | adjusted |
| 2014 | 56 | 0.45 | 0.49 (0.35-0.62) | 56 | 0.59 | 0.58 (0.45-0.72) | 56 | 0.57 | 0.55 (0.42-0.69) |
| 2015 | 130 | 0.34 | 0.34 (0.26-0.42) | 126 | 0.52 | 0.52 (0.43-0.60) | 125 | 0.55 | 0.55 (0.47-0.64) |
| 2016 | 127 | 0.40 | 0.41 (0.32-0.49) | 125 | 0.57 | 0.57 (0.48-0.65) | 122 | 0.62 | 0.63 (0.54-0.71) |
| 2017 | 151 | 0.41 | 0.40 (0.33-0.48) | 151 | 0.62 | 0.61 (0.53-0.69) | 150 | 0.57 | 0.58 (0.50-0.66) |
| 2018 | 155 | 0.40 | 0.40 (0.32-0.48) | 150 | 0.53 | 0.53 (0.45-0.61) | 148 | 0.52 | 0.52 (0.45-0.60) |
| 2019 | 107 | 0.38 | 0.40 (0.31-0.50) | 106 | 0.57 | 0.59 (0.49-0.68) | 98 | 0.58 | 0.58 (0.47-0.67) |
| 2020 | 148 | 0.40 | 0.41 (0.33-0.49) | 133 | 0.59 | 0.58 (0.50-0.67) |  |  |  |
| 2021 | 44 | 0.50 | 0.49 (0.30-0.68) |  |  |  |  |  |  |

| **Supplementary Table 4** Characteristics of patients working and patients without medical benefit prior sepsis admission and discharged alive | | | |
| --- | --- | --- | --- |
|  | **Working**  **n= 12.260 (34.2%)** | **Without medical benefit^a^ n=8.311 (23.1%)** | **P-value** |
| **Characteristics** |  |  |  |
| Male, n (%) | 7 341 (59.9) | 4 475 (53.8) | >0.001 |
| Age, years, mean (SD) | 43.7 (11.8) | 40.5 (13.2) | >0.001 |
| Age-group, n (%) |  |  | >0.001 |
| 18-29 | 2 077 (16.9) | 2 240 (27.0) |  |
| 30-39 | 2 314 (18.9) | 1 605 (19.3) |  |
| 40-49 | 3 121 (25.4) | 1 837 (22.1) |  |
| 50-59 | 4 748 (38.7) | 2 629 (31.6) |  |
| Comorbidities, n (%) |  |  |  |
| Heart and vascular | 2 394 (19.5) | 1 249 (15.0) | >0.001 |
| Cancer | 1 941 (15.8) | 711 (8.6) | >0.001 |
| Lung | 681 (5.6) | 476 (5.7) | 0.598 |
| Diabetes | 666 (5.4) | 547 (6.6) | 0.001 |
| Immune | 269 (2.2) | 165 (2.0) | 0.307 |
| Renal | 145 (1.2) | 122 (1.5) | 0.076 |
| Liver | 44 (0.4) | 36 (0.4) | 0.401 |
| Number of comorbidities, n (%) |  |  | >0.001 |
| 0 | 7 290 (58.5) | 5 601 (67.4) |  |
| 1 | 3 933 (32.1) | 2 193 (26.4) |  |
| 2 | 911 (7.4) | 442 (5.3) |  |
| ≥3 | 126 (1.0) | 75 (0.9) |  |
| Site of infection, n (%) |  |  |  |
| Respiratory | 3 692 (30.2) | 2 694 (32.4) | >0.001 |
| Genitourinary | 1 602 (13.1) | 1 185 (14.3) | 0.014 |
| Skin and soft tissue | 558 (4.4) | 576 (6.9) | >0.001 |
| Gastrointestinal | 827 (6.8) | 485 (6.0) | 0.054 |
| Intra-abdominal | 755 (6.2) | 391(4.7) | >0.001 |
| Infections following a procedure | 625 (5.1) | 374 (4.5) | 0.05 |
| Endocarditis/myocarditis | 190 (1.6) | 125 (1.5) | 0.793 |
| Other^b^ | 2 056 (16.8) | 1 507 (18.2) | 0.011 |
| COVID-19-related sepsis^c^ | 384 (3.1) | 713 (8.6) | >0.001 |
| Organ system with acute dysfunction, n (%) |  |  |  |
| Respiratory | 3 063 (25.0) | 2 204 (26.5) | 0.013 |
| Circulatory | 878 (7.2) | 471 (5.7) | >0.001 |
| Renal | 2 627 (21.4) | 1 697 (20.4) | 0.081 |
| Hepatic | 194 (1.6) | 106 (1.3) | 0.072 |
| Coagulation | 757 (6.2) | 346 (4.2) | >0.001 |
| Other^c^ | 2 543 (20.7) | 1 847 (22.2) | 0.011 |
| Number of acute organ dysfunctions, n (%) |  |  | >0.001 |
| 1 | 6 422 (87.2) | 4 564 (90.2) |  |
| 2 | 736 (10.0) | 398 (7.9) |  |
| 3 | 164 (2.2) | 80 (1.6) |  |
| ≥4 | 42 (0.6) | 18 (0.4) |  |
| ICU treatment^d^, n (%) | 951 (7.8) | 590 (7.1) | 0.079 |
| Length of hospital stay, days, mean (SD) |  |  |  |
| Non-ICU patients | 12.9 (22.2) | 9.2 (13.7) | >0.001 |
| ICU patients^e^ | 25.4 (35.4) | 18.6 (19.8) | >0.001 |
| 30-day Readmission^f^, n (%) | 3 664 (29.9) | 1 854 (22.3) | >0.001 |
| Abbreviation: NA=Not Applicable. ICU= Intensive Care Unit,  ^a^ Sepsis patients registered without a sickness leave 31 days before admission and 31days after admisson  ^b^ Other infections= Bone, obstetric, upper airway, central nervous system and unknown  ^c^ Variable calculated from 28^th^ February 2020  ^d^ Other acute organ dysfunction= Acidosis, unspecific gangrene, central nervous system dysfunctions and Systemic Inflammatory Response Syndrome.  ^e^ Variable calculated from May 1, 2014  ^f^ Readmission= admission within 30 days after discharge regardless of cause | | | |

| **Supplementary Table 5** Associations of patient and clinical characteristics with sustained RTW in ICU and non-ICU sepsis patients | | | | | | | | | | |
| --- | --- | --- | --- | --- | --- | --- | --- | --- | --- | --- |
|  | ICU^a^ | | | | | Non-ICU^b^ | | | | |
| Variable | Person year (py) at risk | Events | Rate per  py | Crude HR | Adjusted^c^ HR (95% CI) | Person year (py) at risk | Events | Rate per  py | Crude HR | Adjusted^c^ HR (95% CI) |
| Age-group |  |  |  |  |  |  |  |  |  |  |
| 18-29 | 75 | 110 | 1.48 | 1.00 | 1.00 (Reference) | 645 | 1 669 | 2.59 | 1.00 | 1.00 (Reference) |
| 30-39 | 74 | 129 | 1.73 | 1.03 | 1.02 (0.80 – 1.31) | 750 | 1 837 | 2.45 | 0.89 | 0.89 (0.83 – 0.95) |
| 40-49 | 101 | 163 | 1.61 | 0.98 | 0.98 (0.77 – 1.25) | 978 | 2 389 | 2.44 | 0.78 | 0.78 (0.73– 0.83) |
| 50-60 | 146 | 258 | 1.77 | 0.85 | 0.85 (0.68 – 1.07) | 1 449 | 3 334 | 2.30 | 0.68 | 0.67 (0.64 – 0.72) |
| Sex |  |  |  |  |  |  |  |  |  |  |
| Male | 279 | 451 | 1.62 | 1.00 | 1.00 (Reference) | 2301 | 5488 | 2.39 | 1.00 | 1.00 (Reference) |
| Female | 117 | 209 | 1.78 | 1.15 | 1.14 (0.87 – 1.34) | 1522 | 3 741 | 2.46 | 1.01 | 0.987 (0.985 – 0.989) |
| Sepsis subgroup^d^ |  |  |  |  |  |  |  |  |  |  |
| Sepsis | 382 | 630 | 1.65 | 1.00 | 1.00 (Reference) | 296 | 848 | 2.86 | 1.00 | 1.00 (Reference) |
| COVID-19-related | 14 | 30 | 2.13 | 1.36 | 1.41 (0.98 – 2.05) | 81 | 294 | 3.63 | 1.20 | 1.23 (1.07 – 1.42) |
| Site of infection^e^ |  |  |  |  |  |  |  |  |  |  |
| Respiratory | 169 | 262 | 1.55 | 1.00 | 1.00 (Reference) | 818 | 2 200 | 2.69 | 1.00 | 1.00 (Reference) |
| Genitourinary | 15 | 19 | 1.24 | 1.09 | 1.07 (0.67 – 1.72) | 245 | 766 | 3.12 | 1.32 | 1.30 (1.20 – 1.42) |
| Intra-abdominal | 9 | 23 | 2.45 | 1.48 | 1.48 (0.96 – 2.28) | 153 | 368 | 2.40 | 0.96 | 0.96 (0.86 – 1.07) |
| Gastrointestinal infections | 4 | 12 | 3.42 | 1.75 | 1.81 (1.01 – 3.24) | 165 | 625 | 3.80 | 1.57 | 1.52 (1.39 – 1.66) |
| Skin and soft tissue | 9 | 13 | 1.48 | 1.97 | 1.90 (1.08– 3.32) | 96 | 263 | 2.74 | 1.16 | 1.18 (1.04 – 1.34) |
| Infections following a procedure | 10 | 17 | 1.67 | 0.95 | 0.97 (0.59 – 1.60) | 125 | 227 | 1.81 | 0.79 | 0.80 (0.69 – 0.91) |
| Endocarditis/myocarditis | 4 | 5 | 1.41 | 1.13 | 1.15 (0.48 – 2.80) | 43 | 69 | 1.61 | 0.65 | 0.64 (0.50 – 0.82) |
| Other infections^f^ | 78 | 142 | 1.81 | 1.26 | 1.24 (1.01 – 1.52) | 715 | 1 658 | 2.32 | 0.95 | 0.91 (0.86 – 0-97) |
| Comorbidities^e^ |  |  |  |  |  |  |  |  |  |  |
| Heart and vascular | 126 | 178 | 1.41 | 1.00 | 1.00 (Reference) | 525 | 997 | 1.90 | 1.00 | 1.00 (Reference) |
| Cancer | 8 | 8 | 1.01 | 0.57 | 0.57 (0.28 – 1.17) | 740 | 728 | 0.98 | 0.52 | 0.52 (0.48 – 0.58) |
| Lung | 17 | 29 | 1.73 | 1.58 | 1.63 (1.09 – 2.40) | 121 | 338 | 2.78 | 1.57 | 1.60 (1.42 – 1.80) |
| Diabetes | 13 | 31 | 2.44 | 2.49 | 2.53 (1.72 – 3.73) | 85 | 238 | 2.81 | 1.58 | 1.58 (1.39 – 1.81) |
| Renal | <1 | 1 | 2.83 | 3.72 | 3.63 (0.50 – 26.27) | 16 | 24 | 1.45 | 0.99 | 0.98 (0.66 – 1.46) |
| Immune | 1.23 | 2 | 1.63 | 1.94 | 2.08 (0.50 – 8.57) | 28 | 97 | 3.50 | 2.07 | 2.06 (1.67 – 2.53) |
| Liver | NA | NA | NA | NA | NA | 5 | 10 | 2.03 | 0.60 | 0.62 (0.33 – 1.15) |
| No. of comorbidities |  |  |  |  |  |  |  |  |  |  |
| 0 | 226 | 373 | 1.65 | 1.00 | 1.00 (Reference) | 1 914 | 6 242 | 3.26 | 1.00 | 1.00 (Reference) |
| 1 | 91 | 133 | 1.45 | 0.71 | 0.73 (0.62 – 0.86) | 1 522 | 2 432 | 1.60 | 0.45 | 0.46 (0.44 – 0.48) |
| 2 | 27 | 37 | 1.39 | 0.58 | 0.59 (0.44 – 0.79) | 348 | 495 | 1.42 | 0.38 | 0.39 (0.36 – 0.42) |
| ≥3 | 7 | 9 | 1.36 | 0.38 | 0.41 (0.13 – 1.27) | 39 | 60 | 1.55 | 0.30 | 0.32 (0.25 – 0.41) |
| Type of acute organ dysfunction^e^ |  |  |  |  |  |  |  |  |  |  |
| Respiratory | 110 | 182 | 1.65 | 1.00 | 1.00 (Reference) | 709 | 1 676 | 2.36 | 1.00 | 1.00 (Reference) |
| Renal | 22 | 54 | 2.46 | 1.91 | 1.93 (1.42 – 2.62) | 541 | 1 707 | 3.15 | 1.36 | 1.38 (1.29 – 1.47) |
| Circulatory | 51 | 87 | 1.70 | 1.39 | 1.39 (1.08 – 1.80) | 136 | 266 | 1.96 | 0.85 | 0.89 (0.78 – 1.01) |
| Coagulation | 2 | 5 | 2.33 | 1.22 | 1.95 (0.43 – 2.58) | 330 | 402 | 1.22 | 0.58 | 0.57 (0.51 – 0.63) |
| Hepatic | 1 | 2 | 1.82 | 0.30 | 0.30 (0.08 – 1.23) | 36 | 64 | 1.78 | 0.85 | 0.83 (0.65 – 1.07) |
| Other acute organ dysfunctions^g^ | 39 | 43 | 1.09 | 0.87 | 0.88 (0.63 –1.23) | 236 | 692 | 2.93 | 1.35 | 1.33 (1.22 – 1.46) |
| No. of acute organ dysfunctions |  |  |  |  |  |  |  |  |  |  |
| 1 | 199 | 356 | 1.79 | 1.00 | 1.00 (Reference) | 1 988 | 4 807 | 2.41 | 1.00 | 1.00 (Reference) |
| 2 | 165 | 249 | 1.50 | 0.92 | 0.93 (0.76 – 1.14) | 246 | 359 | 1.46 | 0.60 | 0.60 (0.55 – 0.66) |
| 3 | 30 | 52 | 1.73 | 1.07 | 1.09 (0.77 – 1.52) | 56 | 72 | 1.29 | 0.57 | 0.56 (0.46 – 0.68) |
| ≥4 | 2 | 3 | 1.62 | 0.79 | 0.78 (0.40 – 1.51) | 11 | 16 | 1.45 | 0.49 | 0.49 (0.33 – 0.72) |
| Abbrevation: HR=Hazard Ratio, CI= Confidence Interval, ICU= Intensive Care Unit, NA= Not Applicable  ^a^ Enter date =May 1, 2014  ^b^ Enter date= July 1, 2010  ^c^ Cox regression with time to death as dependent variable, the listed variable as covariate (one at the time), and sex and age.  ^d^ Enter date=February 28, 2020  ^e^ Categorical variable where one ICD-10 code excludes other ICD-10 codes in the same diagnosis group  ^f^ Other infections= Bone, obstetric, upper airway, central nervous system and unknown  ^g^ Other acute organ dysfunctions= Acidosis, unspecific gangrene, central nervous system dysfunctions and Systemic Inflammatory Response Syndrome. | | | | | | | | | | |

| **Supplementary Table 6** Adjusted hazard ratio from Cox regression by characteristics of patients working in at least 92 consecutive days without sickness benefit after index sepsis admission. | | | | | |
| --- | --- | --- | --- | --- | --- |
| Variable | Person year (py) at risk | Events | Rate per  py | Crude HR | Adjusted^a^ HR (95% C) |
| Age-group |  |  |  |  |  |
| 18-29 | 1 065 | 1 686 | 1.58 | 1.00 | 1.00 (Reference) |
| 30-39 | 1 218 | 1 854 | 1.52 | 0.91 | 0.91 (0.85 – 0.97) |
| 40-49 | 1 548 | 1 548 | 1.53 | 0.82 | 0.82 (0.77 – 0.87) |
| 50-60 | 2 157 | 2 157 | 1.52 | 0.71 | 0.71 (0.67 – 0.75) |
| Sex |  |  |  |  |  |
| Male | 3 605 | 5 493 | 1.52 | 1.00 | 1.00 (Reference) |
| Female | 2 382 | 3 710 | 1.56 | 1.04 | 1.01 (0.97 – 1.06) |
| Sepsis subgroup ^b^ |  |  |  |  |  |
| Sepsis | 280 | 561 | 2.00 | 1.00 | 1.00 (Reference) |
| COVID-19-related | 137 | 298 | 2.18 | 1.31 | 1.35 (1.17 – 1.56) |
| Site of infection^c^ |  |  |  |  |  |
| Respiratory | 1 410 | 2 288 | 1.51 | 1.00 | 1.00 (Reference) |
| Genitourinary | 411 | 743 | 1.81 | 1.36 | 1.34 (1.23 – 1.45) |
| Intra-abdominal | 255 | 364 | 1.61 | 1.05 | 1.05 (0.94 – 1.17) |
| Gastrointestinal infections | 309 | 610 | 1.97 | 1.59 | 1.55 (1.42 – 1.69) |
| Skin and soft tissue | 145 | 263 | 1.82 | 1.27 | 1.29 (1.13 – 1.47) |
| Infections following a procedure | 181 | 227 | 1.25 | 0.82 | 0.83 (0.72 – 0.95) |
| Endocarditis/myocarditis | 58 | 64 | 1.11 | 0.66 | 0.66 (0.51 – 0.84) |
| Other infections^d^ | 1 115 | 1 685 | 1.51 | 1.01 | 0.97 (0.91 – 1.04) |
| Comorbidities^c^ |  |  |  |  |  |
| Heart and vascular | 807 | 1 044 | 1.29 | 1.00 | 1.00 (Reference) |
| Cancer | 822 | 603 | 0.73 | 0.50 | 0.51 (0.46 – 0.56) |
| Lung | 204 | 343 | 1.67 | 1.62 | 1.61 (1.43 – 1.83) |
| Diabetes | 156 | 250 | 1.60 | 1.54 | 1.55 (1.35 – 1.78) |
| Renal | 19 | 20 | 1.03 | 0.91 | 0.90 (0.58 – 1.40) |
| Immune | 50 | 95 | 1.89 | 2.01 | 2.02 (1.64 – 2.50) |
| Liver | 8 | 8 | 1.00 | 0.53 | 0.54 (0.27 – 1.08) |
| No. of comorbidities |  |  |  |  |  |
| 0 | 3 410 | 6 314 | 1.85 | 1.00 | 1.00 (Reference) |
| 1 | 2 067 | 2 363 | 1.14 | 0.44 | 0.45 (0.43 – 0.47) |
| 2 | 460 | 471 | 1.02 | 0.37 | 0.38 (0.34 – 0.42) |
| ≥3 | 50 | 55 | 1.09 | 0.31 | 0.32 (0.25 –0.42) |
| Type of acute organ dysfunction^c^ |  |  |  |  |  |
| Respiratory | 1 113 | 1 722 | 1.54 | 1.00 | 1.00 (Reference) |
| Renal | 905 | 1 676 | 1.85 | 1.42 | 1.43 (1.34 – 1.53) |
| Circulatory | 240 | 325 | 1.36 | 0.89 | 0.92 (0.82 – 1.03) |
| Coagulation | 398 | 356 | 0.89 | 0.60 | 0.59 (0.52 – 0.66) |
| Hepatic | 48 | 57 | 1.20 | 0.78 | 0.77 (0.588 – 0.997) |
| Other acute organ dysfunctions^e^ | 411 | 679 | 1.65 | 1.25 | 1.22 (1.12 – 1.34) |
| No. of acute organ dysfunctions |  |  |  |  |  |
| 1 | 3 114 | 4 815 | 1.54 | 1.00 | 1.00 (Reference) |
| 2 | 387 | 409 | 1.05 | 0.55 | 0.56 (0.51 – 0.62) |
| 3 | 98 | 91 | 0.93 | 0.52 | 0.51 (0.42 – 0.63) |
| ≥4 | 17 | 22 | 1.25 | 0.50 | 0.50 (0.33 – 0.76) |
| ICU treatment^f^ |  |  |  |  |  |
| No | 3 437 | 5 785 | 1.68 | 1.00 | 1.00 (Reference) |
| Yes | 476 | 568 | 1.19 | 0.54 | 0.53 (0.49 – 0.58) |
| Abbrevation: HR=Hazard Ratio, CI= Confidence Interval. ICU= Intensive Care Unit  ^a^ Cox regression with time to death as dependent variable, the listed variable as covariate (one at the time), and sex and age.  ^b^ Enter date=February 27, 2020  ^c^ Categorical variable where one ICD-10 code excludes other ICD-10 codes in the same diagnosis group  ^d^ Other infections= Bone, obstetric, upper airway, central nervous system and unknown  ^e^ Other acute organ dysfunctions= Acidosis, unspecific gangrene, central nervous system dysfunctions and Systemic Inflammatory Response Syndrome.  ^f^ Enter date= May 1, 2014 | | | | | |

| **Supplementary Table 7** Adjusted hazard ratio from Cox regression by characteristics of patients working in at least 31 consecutive days without sickness benefit after index sepsis admission (not mutually exclusive categories) | | | | | |
| --- | --- | --- | --- | --- | --- |
| Variable | Person year (py) at risk | Events | Rate per py | Crude HR | Adjusted HR^a^ (95% CI) |
| Infection site |  |  |  |  |  |
| Respiratory^b^ | 1254 | 2895 | 2.31 | 0.89 | 0.90 (0.86–0.94) |
| Genitourinary^b^ | 465 | 1 371 | 2.95 | 1.30 | 1.31 (1.24–1.39) |
| Intra-abdominal^b^ | 263 | 585 | 2.22 | 0.90 | 0.91 (0.84–0.99) |
| Gastrointestinal infections^b^ | 181 | 560 | 3.01 | 1.33 | 1.31 (1.20–1.42) |
| Skin and soft tissue^b^ | 202 | 468 | 2.32 | 1.03 | 1.04 (0.95–1.14) |
| Infections following a procedure^b^ | 274 | 469 | 1.71 | 0.71 | 0.73 (0.67–0.80) |
| Endocarditis/myocarditis^b^ | 89 | 141 | 1.58 | 0.67 | 0.67 (0.57–0.79) |
| Other infections^b,c^ | 710 | 1 678 | 2.36 | 1.00 | 0.99 (0.94–1.05) |
| Comorbidities |  |  |  |  |  |
| Heart and vascular^d^ | 1 000 | 1 668 | 1.68 | 0.60 | 0.62 (0.59–0.65) |
| Cancer^d^ | 935 | 953 | 1.02 | 0.33 | 0.34 (0.31–0.36) |
| Lung^d^ | 235 | 507 | 2.16 | 0.82 | 0.85 (0.78–0.93) |
| Diabetes^d^ | 234 | 510 | 2.18 | 0.82 | 0.88 (0.80–0.96) |
| Renal^d^ | 68 | 101 | 1.47 | 0.68 | 0.68 (0.56–0.83) |
| Immune^d^ | 91 | 210 | 2.30 | 0.91 | 0.89 (0.78–1.02) |
| Liver^d^ | 11 | 18 | 2.34 | 0.37 | 0.39 (0.24–0.61) |
| Type of acute organ dysfunction |  |  |  |  |  |
| Respiratory^e^ | 1 141 | 2 279 | 2.0 | 0.74 | 0.74 (0.71–0.78) |
| Renal^e^ | 839 | 2 189 | 2.61 | 1.12 | 1.15 (1.10–1.21) |
| Circulatory^e^ | 392 | 647 | 1.64 | 0.66 | 0.68 (0.63–0.74) |
| Coagulation^e^ | 394 | 502 | 1.27 | 0.56 | 0.55 (0.50–0.60) |
| Hepatic^e^ | 84 | 123 | 1.46 | 0.60 | 0.59 (0.50–0.71) |
| Other acute organ dysfunctions^e,f^ | 819 | 2 169 | 2.65 | 1.19 | 1.18 (1.13–1.24) |
| Abbrevation: HR=Hazard Ratio, CI= Confidence Interval.  ^a^ Cox regression with time to death as dependent variable, the listed variable as covariate (one at the time), adjusted for sex and age.  ^b^ Reference group= All other infection sites  ^c^ Other infections= Bone, obstetric, upper airway, central nervous system and unknown  ^d^ Reference group= All other comorbidities  ^e^ Reference group= All other acute organ dysfunctions  ^f^ Other acute organ dysfunctions= Acidosis, unspecific gangrene, central nervous system dysfunctions and Systemic Inflammatory Response Syndrome. | | | | | |

# References

1. Bakken IJ, Ariansen AMS, Knudsen GP, Johansen KI, Vollset SE. The Norwegian Patient Registry and the Norwegian Registry for Primary Health Care: Research potential of two nationwide health-care registries. *Scand J Public Health*. Feb 2020;48(1):49-55. doi:10.1177/1403494819859737

2. ICD-10 og ICD-11. Directorate of e-health. Updated April 2022. Accessed December 15, 2022. <https://www.ehelse.no/kodeverk-og-terminologi/ICD-10-og-ICD-11>

3. Norwegian Intensive Registry. <https://helse-bergen.no/norsk-intensivregister-nir>

4. NAV. Membership of the National Insurance Scheme Accessed April 15 2023. <https://www.nav.no/en/home/rules-and-regulations/membership-of-the-national-insurance-scheme>

5. Norwegian Cause of Death Registry. <https://helsedata.no/en/forvaltere/norwegian-institute-of-public-health/norwegian-cause-of-death-registry/>

6. Skei NV, Nilsen TIL, Knoop ST, et al. Long-term temporal trends in incidence rate and case fatality of sepsis and COVID-19-related sepsis in Norwegian hospitals, 2008-2021: a nationwide registry study. *BMJ Open*. Aug 2 2023;13(8):e071846. doi:10.1136/bmjopen-2023-071846
